# Associations of Serum Urate and Cardiovascular Events in a Clinical Trial of Interleukin-1β Blockade

**DOI:** 10.1016/j.jacadv.2024.101583

**Published:** 2025-01-24

**Authors:** Nicholas H. Adamstein, Jean G. MacFadyen, Brittany N. Weber, Peter Libby, Daniel H. Solomon, Paul M Ridker

**Affiliations:** a Division of Cardiovascular Medicine, Department of Medicine, Brigham and Women’s Hospital, Harvard Medical School, Boston, Massachusetts, USA; b Center for Cardiovascular Disease Prevention, Divisions of Preventive Medicine and Cardiovascular Diseases, Brigham and Women’s Hospital, Harvard Medical School, Boston, Massachusetts, USA; c Division of Rheumatology, Inflammation, and Immunity, Brigham and Women’s Hospital, Harvard Medical School, Boston, Massachusetts, USA

**Keywords:** serum urate, atherosclerosis, major adverse cardiovascular events, inflammation, cytokines, gout

## Abstract

**Background:**

Serum urate (SU) associates with cardiovascular (CV) events, mortality, and gout.

**Objectives:**

The purpose of this study was to assess whether SU predicts CV risk in a trial of interleukin (IL)-1β inhibition with canakinumab, and whether IL-1β blockade, kidney function, or gout alter these associations.

**Methods:**

This study is a subanalysis of the Canakinumab Antiinflammatory Thrombosis Outcome Study (CANTOS), which randomized 10,061 patients with prior myocardial infarction and elevated high-sensitivity C-reactive protein to 3 doses of canakinumab or placebo. SU was measured at baseline. Cox proportional hazards models compared major adverse cardiovascular events (MACE), CV death, and all-cause mortality among those with SU ≤6.8 mg/dL (normal), 6.8 to 9.0 mg/dL (elevated), and ≥9.0 mg/dL (markedly elevated). Cox regressions were repeated within subgroups, including canakinumab vs placebo, estimated glomerular filtration rate ≥60 vs <60 mL/min, and gout vs no gout.

**Results:**

Markedly elevated SU associated with MACE (HR: 1.66 [95% CI: 1.38-1.99]; *P* < 0.0001), CV death (HR: 2.52 [95% CI: 1.98-3.21]; *P* < 0.0001), and all-cause mortality (HR: 2.43 [95% CI: 2.01-2.94]; *P* < 0.0001) compared to normal SU. After multivariable adjustment for a minimal set of potential confounders, SU independently predicted all 3 endpoints. Associations were unchanged after IL-1β blockade with canakinumab. For normal estimated glomerular filtration rate, SU associated with CV and all-cause mortality, but not MACE. Participants with gout had higher event rates independent of SU.

**Conclusions:**

In over 10,000 patients with coronary artery disease, individuals with markedly elevated SU have elevated CV risk despite aggressive treatment. IL-1β blockade did not modify these associations. Baseline kidney function and monosodium urate deposition may function as effect modifiers.

Uric acid, a byproduct of purine metabolism and the central metabolite in gout pathogenesis, associates with cardiovascular (CV) events and mortality.^1^, ^2^, ^3^ However, patients with gout and hyperuricemia commonly have other risk factors for atherosclerotic cardiovascular disease (ASCVD), such as advanced age, hypertension, obesity, metabolic syndrome, obstructive sleep apnea, alcohol use, and kidney disease, which questions whether hyperuricemia is causally related to CV events, or merely associated through confounding bias.

Mendelian randomization studies have attempted to identify causal relationships between SU, gout, and atherosclerosis with inconsistent results. While some studies have found associations between genetic determinants of SU and atherosclerotic risk,^4^^,^^5^ others have been unable to support a causal role for SU in CV disease.^6^^,^^7^ One of these studies identified loci linking hyperuricemia—but not gout—to atherosclerotic events.^4^ It remains uncertain whether gout is relevant to the observed relationships between SU and atherosclerosis. Based on observational data, the risk of CV disease appears to be higher in patients with hyperuricemia and gout, than in those with just hyperuricemia,^4^ and a case-control study found a higher odds of recent gout flares in participants who suffered a myocardial infarction (MI) than in those who did not.^8^

The NLRP3 inflammasome pathway of innate immunity has been implicated in the pathophysiology of both gout and ASCVD.^9^, ^10^, ^11^ The inflammasome and downstream cytokines have thus become a therapeutic target for both diseases. CANTOS (Canakinumab Anti-inflammatory Thrombosis Outcome Study) was the first randomized controlled trial to demonstrate that targeting the NLRP3 inflammasome pathway via interleukin (IL)-1β blockade reduced incident major adverse cardiovascular events (MACE).^12^ A follow-up analysis found that canakinumab also reduced rates of incident gout regardless of baseline SU, and with no effect on SU levels.^13^

Using data from CANTOS, we validated associations between hyperuricemia and atherosclerotic events and mortality in over 10,000 participants with CV disease and aggressively treated with guideline-directed therapies. Given the common inflammatory pathway implicated in gout and ASCVD, we used this unique data set to quantify associations between SU and CV events in the setting of IL-1β blockade. To address effect modification by kidney function, we repeated the same analyses stratified by estimated glomerular filtration rate (eGFR). Finally, we stratified by baseline diagnosis of gout to assess the relevance of monosodium urate (MSU) deposition to these associations.

## Methods

### Cantos trial design

We performed a subanalysis of the CANTOS, which randomized 10,061 participants aged 18 years or older with a history of prior MI (at least 30 days prior to randomization) and a baseline high-sensitivity C-reactive protein (hsCRP) level ≥2 mg/L to canakinumab 50 mg, 150 mg, 300 mg, or placebo, administered subcutaneously every 3 months.^12^ Patients with chronic or recurrent infections, previous cancer (other than basal-cell skin carcinoma), immunocompromised states, high-risk tuberculosis, or use of systemic anti-inflammatory treatments were excluded. Participants were recruited from 39 countries between April 28, 2011, and March 3, 2014, and were followed for a median of 3.7 years and up to 4 years (last study visit June 2, 2017) for a primary composite endpoint of three-point MACE comprised of nonfatal MI, nonfatal stroke, and CV death. All participants, including those who discontinued study therapy, were followed until death or the end of the study, and the investigators aimed to determine the vital status of all participants at the final visit.^12^ CANTOS protocol was approved by the Institutional Review Board and Independent Ethics Committee prior to study initiation. All participants gave written informed consent to participate in CANTOS and for their data to be used in follow-up analyses. The CANTOS sponsor had input on the design of the trial protocol and was responsible for data collection. Detailed methods including trial design, outcomes assessment, measurement of covariables, and handling of missing data are available in the original CANTOS report and its supplements.^12^

### Primary analyses

SU levels were obtained at the time of randomization and at 3-, 6-, 9-, 12-, 24-, 36-, and 48-month follow-up visits. Cox proportional hazards regression models, stratified by time since index MI, compared the hazard of MACE, CV death, and all-cause mortality across 3 baseline SU groups: SU ≤6.8 mg/dL (normal), 6.8 to 9.0 mg/dL (elevated), and ≥9.0 mg/dL (markedly elevated), which were chosen based on established clinical cut points according to the American College of Rheumatology Guidelines.^14^ Inclusive multivariable models adjusted for a wide range of covariables, including randomization arm, age, sex, body mass index (BMI), smoking status, hypertension, diabetes, low-density lipoprotein cholesterol, hsCRP, eGFR, diuretic use, urate-lowering therapy, daily alcohol use, and daily exercise. In a sensitivity analysis, we removed 1 covariable at a time from the multivariable model to identify those with the largest impact on the magnitude of associations. Parsimonious multivariable models adjusted for a minimum set of the most likely confounders that are known to influence both SU levels and CV risk, including age, sex, BMI, diabetes, smoking, daily alcohol use, and daily exercise.

### Subgroup analyses

The Cox regression models were repeated separately within key subgroups: canakinumab versus placebo, eGFR ≥60 mL/min versus <60 mL/min, and baseline gout versus no gout. When the sample was divided by eGFR and baseline gout, the models were adjusted only for treatment arm. When the sample was divided by treatment arm, the models were unadjusted.

Cox proportional hazards models were used to compare the risk of MACE, CV death, and all-cause mortality between those with versus without gout, as well as those who did versus did not develop an incident gout flare during the trial.

### Serum urate trajectory and correlations

We characterized the distribution and trajectory of SU in the CANTOS cohort. Box-and-whisker plots were used to follow the distribution of SU over time. Spearman correlation coefficients quantified associations between SU and traditional and inflammatory risk markers in all participants, as well as those with and without gout.

### Statistical analyses

All *P* values are 2-sided, and all CIs were computed at the 95% level. The proportional hazards assumption was assessed using the supremum test each time a cox proportional hazards model was used. All statistical analyses were performed using SAS, version 9.4 (SAS institute). The corresponding author had full access to the data used in the study and takes responsibility for its integrity, performed the data analysis, prepared the written draft of the manuscript, and made the decision to submit the manuscript for publication. This study was performed at Brigham and Women’s Hospital in Boston, Massachusetts.

## Results

Baseline characteristics of CANTOS participants in whom baseline SU was measured are included in Table 1 (divided by baseline SU group) and Supplemental Table 1 (divided by randomization group). Baseline SU was measured in 10,059 participants (all except for 2). The median age was 61 years, 25.7% of participants were female, 7.6% had gout, 40% had diabetes, 79.6% had hypertension, 23.5% were current smokers, 3.9% used alcohol daily, 6.99% were taking urate-lowering therapy, 36% were taking diuretics, and 91% were taking statins. The median SU was 6.10 mg/dL, eGFR 79.0 mL/min, and hsCRP 4.20 mg/L. Characteristics were balanced across randomization groups. Participants with markedly elevated SU were numerically more likely to be male, hypertensive, and diabetic. They had lower eGFR and higher hsCRP. They were also more likely to use alcohol daily or take diuretics. The baseline SU in subgroups is shown in Supplemental Figure 1. A trivial number of participants (0.27% of both the placebo and canakinumab arms) had unknown vital status at the end of the study.

**Table 1:** Baseline Characteristics Across Serum Urate Groups

	SU ≤6.8 mg/dL (n = 6,940)	SU 6.8-9.0 mg/dL (n = 2,515)	SU ≥9.0 mg/dL (n = 604)
Placebo	2,326 (33.5%)	831 (33.0%)	186 (30.8%)
Canakinumab 50 mg	1,493 (21.5%)	537 (21.4%)	139 (23.0%)
Canakinumab 150 mg	1,562 (22.5%)	591 (23.5%)	131 (21.7%)
Canakinumab 300 mg	1,559 (22.5%)	556 (22.1%)	148 (24.5%)
Age (y)	61 (54-68)	61 (55-68)	63 (56-70)
Female	2,045 (29.5%)	425 (16.9%)	116 (19.2%)
Current smoking	1,744 (25.1%)	532 (21.2%)	90 (14.9%)
Body mass index (kg/m^2^)	29.4 (26.3-33.3)	30.7 (27.3-34.7)	31.1 (27.5-35.6)
Hypertension	5,410 (78.0%)	2076 (82.5%)	520 (86.1%)
Diabetes	2,758 (39.7%)	973 (38.7%)	296 (49.0%)
Gout	376 (5.42%)	250 (9.94%)	136 (22.5%)
Alcohol use (≥1 per day)	236 (3.40%)	128 (5.09%)	31 (5.14%)
Daily exercise	1,228 (18.4%)	400 (16.4%)	89 (15.3%)
Diuretic use	2076 (29.9%)	1,132 (45.0%)	412 (68.2%)
Statin use	6,366 (91.7%)	2,262 (89.9%)	527 (87.3%)
Urate-lowering therapy	459 (6.61%)	178 (7.08%)	66 (10.9%)
eGFR (mL/min)	82 (69-96)	72 (59-87)	60 (46-73)
SU (mg/dL)	5.55 (4.88-6.22)	7.60 (7.23-8.07)	9.75 (9.25-10.46)
hsCRP (mg/L)	4.03 (2.70-6.70)	4.40 (2.95-7.30)	5.25 (3.35-9.75)
IL-6 (ng/L)	2.49 (1.72-3.92)	2.75 (1.91-4.27)	3.41 (2.24-6.03)
Total cholesterol (mg/dL)	159 (135-188)	161 (139-190)	164 (135-197)
LDL cholesterol (mg/dL)	82 (63-107)	83 (65-106)	83 (62-111)
HDL cholesterol (mg/dL)	44.9 (37.5-53.4)	43 (36-50)	41 (34-48)
Triglycerides (mg/dL)	133 (97-188)	152 (110-208)	164 (116-236)

Values are n (%) or median (IQR).

eGFR = estimated glomerular filtration rate; HDL = high-density lipoprotein; hsCRP = high-sensitivity C-reactive protein; IL = interleukin; LDL = low-density lipoprotein; n = number; SU = serum urate.

Participants with markedly elevated SU had a significantly higher hazard of MACE, CV death, and all-cause mortality compared to those with normal SU. The HRs comparing the markedly elevated SU group to the normal SU group for MACE, CV death, and all-cause mortality were 1.66 (95% CI: 1.38-1.99; *P* < 0.0001), 2.52 (95% CI: 1.98-3.21; *P* < 0.001), and 2.43 (95% CI: 2.01-2.94; *P* < 0.0001) respectively (Central Illustration). Adjustment for treatment arm, age, sex, BMI, smoking status, hypertension, diabetes, low-density lipoprotein cholesterol, hsCRP, eGFR, diuretic use, urate-lowering therapy, daily alcohol use, and daily exercise substantially attenuated the associations, though SU continued to predict CV death and all-cause mortality (Table 2). In a sensitivity analysis, removal of either sex, eGFR, or diuretic use from the model had the largest impact on the magnitude of associations and restored statistical significance to the association between SU and MACE (Supplemental Table 2). In the parsimonious model, which was adjusted for age, sex, BMI, smoking status, diabetes, daily alcohol use, and daily exercise, SU independently predicted all 3 endpoints (Table 2).

**Table 2:** Hazard of MACE, CV Death, and All-Cause Mortality Across Baseline Serum Urate Groups

	SU ≤6.8 mg/dL (n = 6,940)	SU 6.8-9.0 mg/dL (n = 2,515)	SU ≥9.0 mg/dL (n = 604)	Trend
MACE				
Number of events (%)	958 (13.8%)	402 (16.0%)	130 (21.5%)	
Univariable	1.00 (ref)	1.16 (1.03-1.30) *P* = 0.01	1.66 (1.38-1.99) *P* < 0.0001	1.24 (1.14-1.34) *P* < 0.0001
Inclusive multivariable	1.00 (ref)	1.02 (0.90-1.16) *P* = 0.74	1.22 (1.00-1.49) *P* = 0.05	1.07 (0.98-1.17) *P* = 0.12
Parsimonious multivariable	1.00 (ref)	1.14 (1.01-1.28) *P* = 0.03	1.56 (1.29-1.88) *P* < 0.0001	1.21 (1.11-1.31) *P* < 0.0001
Cardiovascular death				
Number of events (%)	386 (5.6%)	201 (8.0%)	80 (13.3%)	
Univariable	1.00 (ref)	1.44 (1.21-1.71) *P* < 0.0001	2.52 (1.98-3.21) *P* < 0.0001	1.54 (1.38-1.72) *P* < 0.0001
Inclusive multivariable	1.00 (ref)	1.26 (1.04-1.51) *P* = 0.02	1.69 (1.29-2.21) *P* = 0.0001	1.29 (1.14-1.46) *P* < 0.0001
Parsimonious multivariable	1.00 (ref)	1.46 (1.22-1.74) *P* < 0.0001	2.36 (1.84-3.03) *P* < 0.0001	1.51 (1.35-1.70) *P* < 0.0001
All-cause mortality				
Number of events (%)	645 (9.3%)	306 (12.2%)	129 (21.4%)	
Univariable	1.00 (ref)	1.31 (1.14-1.50) *P* = 0.0001	2.43 (2.01-2.94) *P* < 0.0001	1.48 (1.36-1.62) *P* < 0.0001
Inclusive multivariable	1.00 (ref)	1.13 (0.97-1.31) *P* = 0.11	1.67 (1.35-2.06) *P* < 0.0001	1.24 (1.13-1.37) *P* < 0.0001
Parsimonious multivariable	1.00 (ref)	1.29 (1.12-1.48) *P* = 0.0005	2.25 (1.85-2.74) *P* < 0.0001	1.43 (1.31-1.57) *P* < 0.0001

Values are n (%) or HR (95% CI). Inclusive multivariable models adjusted for randomization arm, age, sex, body mass index, current smoking status, hypertension, type 2 diabetes, low-density lipoprotein cholesterol, high-sensitivity C-reactive protein, estimated glomerular filtration rate, diuretic use, urate-lowering therapy, daily alcohol use, and daily exercise. Parsimonious multivariable models adjusted for age, sex, body mass index, current smoking status, type 2 diabetes, daily alcohol use, and daily exercise.

CV = cardiovascular; MACE = major adverse cardiovascular events; ref = reference; other abbreviations as in Table 1.

**Figure 1:**
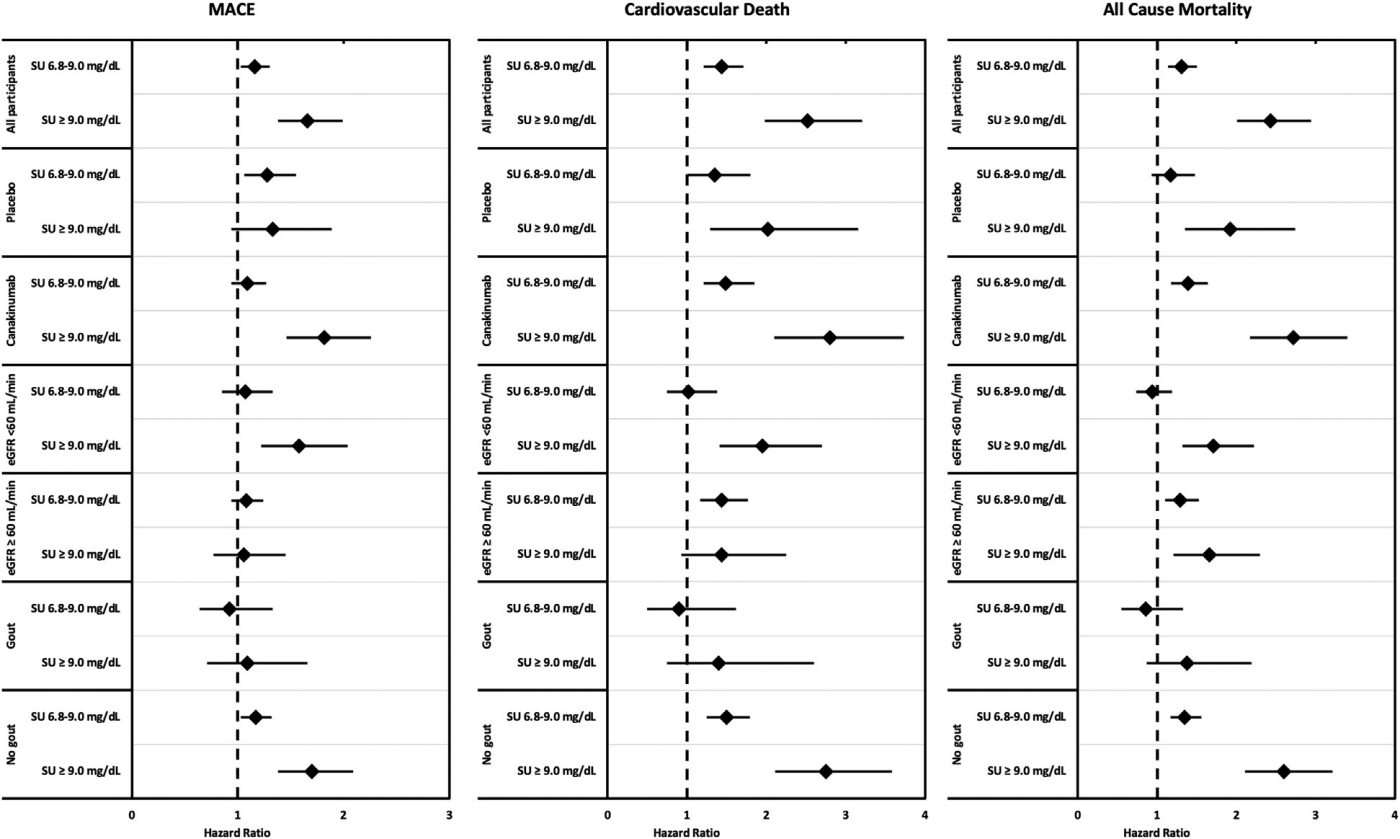
Associations between Serum Urate and Cardiovascular Risk in Key Subgroups Forest plots depicting HRs for MACE (Left), cardiovascular death (Middle), and all-cause mortality (Right) in baseline serum urate groups of 6.8 to 9.0 mg/dL (elevated) and ≥9.0 mg/dL (markedly elevated) compared to a reference group with serum urate ≤6.8 mg/dL (normal). Data are shown separately for all participants, the placebo arm, the canakinumab arm, eGFR <60 mL/Min, eGFR ≥60 mL/Min, those with gout at baseline, and those without gout at baseline. Error bars correspond to 95% CIs. Models were only adjusted for treatment arm except for canakinumab and placebo subgroups, in which case models were unadjusted. Markedly elevated serum urate associates with increased risk of mace, cv death, and all-cause mortality. Associations were unchanged after IL-1β blockade with canakinumab. For those with normal eGFR, ascending SU associates with cardiovascular death and all-cause mortality, but not MACE. SU does not predict cv events or mortality in participants with gout. CV = cardiovascular; eGFR = estimated glomerular filtration rate; MACE = major adverse cardiovascular events; SU = serum urate.

IL-1β blockade did not change the associations between SU and CV outcomes. In the canakinumab arm, the HRs comparing the markedly elevated SU group to the normal SU group were 1.82 (95% CI: 1.46-2.26; *P* < 0.0001), 2.80 (95% CI: 2.10-3.73; *P* < 0.0001), and 2.72 (95% CI: 2.17-3.40; *P* < 0.0001) for MACE, CV death, and all-cause mortality, respectively (Table 3, Figure 1). In analyses stratified by eGFR, SU predicted all 3 composite events in the low eGFR group, and CV death and all-cause mortality, but not MACE, in the normal eGFR group (Table 3, Figure 1). Markedly elevated SU was associated with MACE, CV death, and all-cause mortality in participants without a prior gout diagnosis. However, the associations between SU and all 3 endpoints were extinguished in participants with baseline gout (Table 3, Figure 1).

**Table 3:** Associations Between Serum Urate and Cardiovascular Risk in Key Subgroups

	SU ≤6.8 mg/dL (n = 6,940)	SU 6.8-9.0 mg/dL (n = 2,515)	SU ≥9.0 mg/dL (n = 604)	Trend
MACE				
Placebo	1.00 (ref)	1.28 (1.06-1.55) *P* = 0.01	1.33 (0.94-1.89) *P* = 0.10	1.21 (1.05-1.38) *P* = 0.007
Canakinumab	1.00 (ref)	1.09 (0.94-1.27) *P* = 0.24	1.82 (1.46-2.26) *P* < 0.0001	1.25 (1.14-1.38) *P* < 0.0001
eGFR <60 mL/min	1.00 (ref)	1.07 (0.85-1.33) *P* = 0.58	1.58 (1.22-2.04) *P* = 0.0004	1.23 (1.08-1.40) *P* = 0.002
eGFR ≥60 mL/min	1.00 (ref)	1.08 (0.94-1.24) *P* = 0.27	1.06 (0.77-1.45) *P* = 0.73	1.06 (0.95-1.18) *P* = 0.32
No gout	1.00 (ref)	1.17 (1.03-1.32) *P* = 0.01	1.70 (1.38-2.09) *P* < 0.0001	1.25 (1.14-1.36) *P* < 0.0001
Gout	1.00 (ref)	0.92 (0.64-1.33) *P* = 0.67	1.09 (0.71-1.66) *P* = 0.71	1.02 (0.83-1.26) *P* = 0.85
Cardiovascular death				
Placebo	1.00 (ref)	1.35 (1.01-1.80) *P* = 0.04	2.02 (1.29-3.16) *P* = 0.002	1.39 (1.15-1.70) *P* = 0.0009
Canakinumab	1.00 (ref)	1.49 (1.21-1.85) *P* = 0.0002	2.80 (2.10-3.73) *P* < 0.0001	1.62 (1.42-1.86; *P* < 0.0001
eGFR <60 mL/min	1.00 (ref)	1.02 (0.75-1.38) *P* = 0.91	1.95 (1.41-2.70) *P* < 0.0001	1.36 (1.14-1.61) *P* = 0.0005
eGFR ≥60 mL/min	1.00 (ref)	1.44 (1.17-1.77) *P* = 0.0005	1.44 (0.93-2.25) *P* = 0.10	1.32 (1.13-1.54) *P* = 0.0006
No gout	1.00 (ref)	1.50 (1.25-1.79) *P* < 0.0001	2.75 (2.11-3.58) *P* < 0.0001	1.60 (1.42-1.81) *P* < 0.0001
Gout	1.00 (ref)	0.90 (0.50-1.62) *P* = 0.73	1.40 (0.75-2.60) *P* = 0.29	1.14 (0.83-1.57) *P* = 0.41
All-cause mortality				
Placebo	1.00 (ref)	1.17 (0.93-1.48) *P* = 0.18	1.92 (1.35-2.74) *P* = 0.0003	1.30 (1.11-1.53) *P* = 0.001
Canakinumab	1.00 (ref)	1.39 (1.18-1.64) *P* = 0.0001	2.72 (2.17-3.40) *P* < 0.0001	1.57 (1.42-1.75) *P* < 0.0001
eGFR <60 mL/min	1.00 (ref)	0.94 (0.74-1.19) *P* = 0.60	1.71 (1.32-2.22) *P* < 0.0001	1.26 (1.10-1.44) *P* = 0.0009
eGFR ≥60 mL/min	1.00 (ref)	1.29 (1.10-1.53) *P* = 0.003	1.66 (1.21-2.30) *P* = 0.002	1.29 (1.14-1.46) *P* < 0.0001
No gout	1.00 (ref)	1.35 (1.17-1.56) *P* < 0.0001	2.60 (2.11-3.21) *P* < 0.0001	1.52 (1.38-1.67) *P* < 0.0001
Gout	1.00 (ref)	0.86 (0.55-1.33) *P* = 0.49	1.38 (0.87-2.19) *P* = 0.17	1.13 (0.89-1.43) *P* = 0.32

Values are as HR (95% CI). Models were only adjusted for treatment arm, except for in the canakinumab and placebo subgroups, in which case models were unadjusted.

Abbreviations as in Tables 1 and 2.

**Central Illustration:**
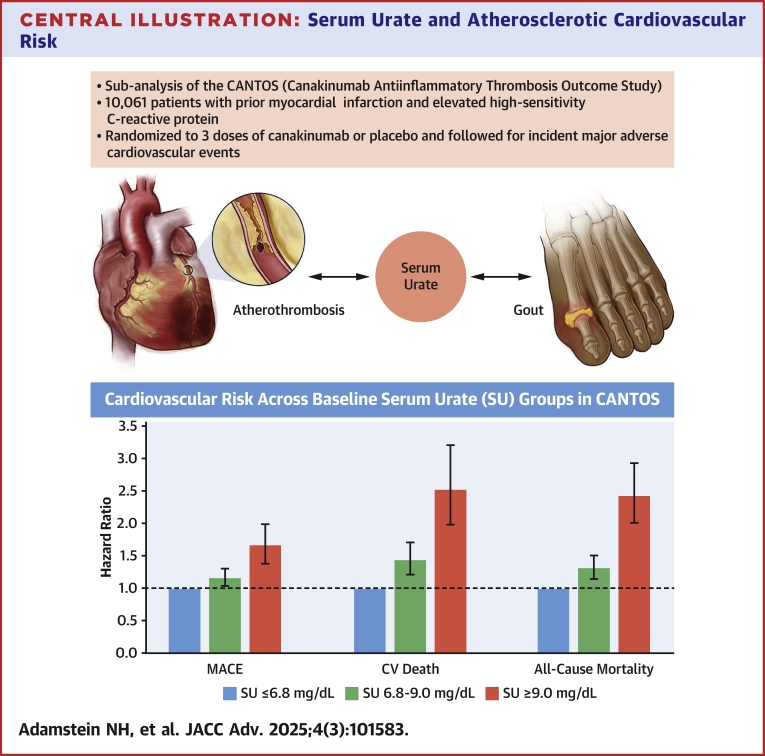
**Serum Urate and Atherosclerotic Cardiovascular Risk** Uric acid, the central metabolite in gout pathogenesis, associates with cardiovascular risk through unknown mechanisms. In CANTOS, elevated baseline serum urate predicts increased risk of MACE, CV death, and all-cause mortality. Error bars correspond to 95% CIs. Abbreviations as in Figure 1.

Participants with baseline gout had significantly higher rates of MACE, CV death, and all-cause mortality compared to those without gout with HRs of 1.46 (95% CI: 1.23-1.72; *P* < 0.0001), 1.31 (95% CI: 1.01-1.70; *P* = 0.04), and 1.45 (95% CI: 1.20-1.76; *P* = 0.0002) respectively. Event rates in participants with an incident gout flare during the trial were numerically but nonsignificantly higher than those who did not develop gout. These data are summarized in Supplemental Table 3.

Baseline SU had a moderate inverse correlation with eGFR (ρ = −0.31) but correlated either weakly or nonsignificantly with inflammatory biomarkers including hsCRP, IL-6, IL-18, white blood cell count, absolute neutrophil count, absolute lymphocyte count, neutrophil-lymphocyte ratio, and fibrinogen (Supplemental Table 4). The correlations were similar in participants with and without gout. SU levels remained stable over 48 months and there was no difference between the placebo and treatment arms, a finding which has been previously reported (Supplemental Figure 2).^13^

## Discussion

Markedly elevated SU predicts MACE, CV death, and all-cause mortality. After adjustment for an inclusive set of covariables that may confound associations between SU and CV risk, markedly elevated SU still independently predicted CV death and all-cause mortality. However, this inclusive multivariate analysis extinguished the association between SU and MACE, with the most impactful covariables being eGFR, diuretic use, and sex. In the parsimonious model, which only adjusted for a minimum set of the most likely confounders with established effects on both SU and CV events, SU independently predicted all 3 endpoints. IL-1β blockade, which reduced incident gout and atherosclerotic events, did not attenuate these associations. When stratifying by eGFR, participants with normal kidney function had significant associations between ascending SU and CV death and all-cause mortality, but not MACE. Participants with gout had increased CV risk compared to those without gout, but their risk was not associated with baseline SU. This study presents a large-scale validation of prior observations of associations between SU and CV events and mortality in over 10,000 participants and builds on these findings by leveraging this robust and unique data set to explore novel mechanistic questions.

These results raise an important question: why do we observe associations between SU and CV events and mortality? Broadly speaking, the relationships are either due to direct causation, indirect causation through an intermediary, or confounding by shared comorbid conditions or risk factors. A direct causal relationship would be best assessed in a randomized controlled trial. A small randomized controlled trial in patients with chronic kidney disease found a significant reduction in broadly defined CV events (inclusive of coronary artery, cerebrovascular, and peripheral arterial endpoints) in participants randomly allocated to allopurinol compared to usual care.^15^ However, the ALL-HEART study, the largest CV outcomes trial to date of urate-lowering therapy, did not identify a reduction in CV events in those randomly allocated to allopurinol.^16^ Of note, the baseline SU of trial participants was in the normal range, there were few participants with kidney disease, and participants with gout were excluded, further limiting the number of patients with hyperuricemia. Moreover, ALL-HEART did not utilize a placebo control, and 59% of participants allocated to allopurinol either never received or discontinued treatment before the end of the study. These considerations prevent us from drawing a definitive conclusion regarding a causal link between hyperuricemia and ASCVD in ALL-HEART.

The present study cannot address a causal relationship between SU and CV events. While the data derived from a randomized controlled trial, participants were not randomized to the 3 baseline SU groups and had different baseline characteristics. While the adjusted models accounted for many confounders, residual confounding is hard to rule-out. However, we believe the data presented in the current study has bearing on the plausibility of several hypothesized causal mechanisms between hyperuricemia and ASCVD.

First, there is prior evidence that hyperuricemia links causally to hypertension.^1^ For example, hyperuricemia predicts increased risk of development of hypertension, often preceding it, and is found more often in patients with primary hypertension than secondary hypertension.^17^ Animal experiments have demonstrated that uricase inhibition leads to elevations of SU as well as development of hypertension.^18^ In the same study, withdrawal of uricase inhibition or treatment with xanthine oxidase inhibition led to improvement in blood pressures. A small randomized controlled trial in adolescents found that allopurinol reduced blood pressure compared to placebo and an observational study in adults found modest reductions in blood pressure with allopurinol use compared to matched controls.^19^^,^^20^ In our study, SU strongly associated with CV events even after adjustment for hypertension, suggesting that there may be other mechanisms at play.

Second, hyperuricemia may lead to kidney disease. Some studies have suggested that treatment of hyperuricemia can delay disease progression and improve kidney function.^21^, ^22^, ^23^ Withdrawal of xanthine oxidase inhibition was shown to increase the risk of progression of kidney disease and worsening of hypertension.^24^ However, larger contemporary randomized controlled trials of allopurinol in patients with chronic kidney disease failed to show reduced decline in eGFR compared to placebo.^25^^,^^26^ The FREED trial found a significant reduction in a composite endpoint of cerebral, CV, and renal events and all-cause mortality in participants randomized to febuxostat compared to usual care, with the benefit largely being driven by reductions in renal impairment.^27^ There were also numerical (but statistically nonsignificant) reductions in nonfatal coronary artery disease, heart failure requiring hospitalization, and arteriosclerotic disease requiring treatment. In our study, adjustment for eGFR substantially attenuated the magnitude of associations between SU and CV events, which would be consistent with either: 1) confounding by kidney disease; or 2) indirect causation with kidney disease as an intermediary. We also note that ascending SU associated with CV death and all-cause mortality even in individuals with normal kidney function. This would suggest that the observed associations between SU and atherosclerotic events do not depend wholly on the presence of impaired kidney function.

Third, a relationship between SU and atherosclerotic events may be due to NLRP3 inflammasome-related inflammation in response to MSU crystal deposition. This inflammatory response mediates many clinical manifestations of gout.^9^ As such, blockade of the NLRP3 inflammasome with canakinumab reduced incident gout without altering SU levels.^13^ The same inflammatory pathway participates in atherothrombosis, and this is the hypothesized reason canakinumab reduced CV events as well.^10^^,^^12^ In both diseases, there is thought to be a similar inflammatory response to crystal deposition (cholesterol crystals in the case of ASCVD, and MSU crystals in the case of gout).^28^ MSU crystals have since been detected in atherosclerotic plaques using dual-energy computed tomography.^29^^,^^30^ One study found that 91.9% of participants with gout had MSU positive plaques, compared to 2.9% of participants with asymptomatic hyperuricemia and 0.38% of controls.^29^ Another study yielded similar results, with pathologic verification using samples from cadavers.^30^ However, the data from our study argue against an IL-1β dependent process. Participants that received IL-1β blockade with canakinumab still had increased risk of MACE, CV death, and all-cause mortality when SU was markedly elevated. This finding provides evidence against the hypothesis that SU is related to atherosclerotic events either by inducing local inflammasome activation within a plaque, or a systemic inflammatory reaction when it deposits in the joints, for example. Interestingly, elevated or markedly elevated SU was not associated with increased event rates in participants with a prior diagnosis of gout. Moreover, participants with gout had a significantly higher hazard of MACE, CV death, and all-cause mortality than those without gout. These observations together suggest that MSU deposition may be an important intermediary in the link between SU and CV risk. There may still be an inflammatory reaction to MSU in the setting of IL-1β inhibition, as canakinumab is known to have no effect on IL-18, a parallel cytokine involved in the NLRP3 inflammasome pathway. Indeed, patients treated with canakinumab continued to have increased risk with higher IL-18 levels.^31^

Finally, hyperuricemia might associate with ASCVD through multiple concurrent and overlapping mechanisms, none of which are necessary or sufficient on their own. In this manner, hyperuricemia may lead to development of hypertension, kidney disease, or MSU deposition disease which may promote atherogenesis to varying degrees in each person.^1^ If this is the case, associations between SU and atherosclerotic events may still be attenuated in multivariable models, even if SU links indirectly to ASCVD.

### Study Limitations

First, our analyses were not prespecified at the time of CANTOS design and should therefore be viewed as exploratory. While our data affirm the established relationship between SU and CV events and mortality, they extend this to an aggressively treated secondary prevention cohort. The relationships we observed might differ in healthy individuals. Finally, as previously stated, our data should not be interpreted as establishing causation, as participants were not randomized to baseline SU groups, and multivariable models can only adjust for known and measured confounders.

It remains uncertain whether SU should become a therapeutic target in ASCVD. While current observational data are consistent,^2^^,^^3^ the largest randomized controlled trial did not support this approach, albeit in participants with normal SU, no history of gout, and predominately preserved kidney function.^16^ Additional large, randomized, placebo-controlled CV outcomes trials in participants with both normal and impaired kidney function would be needed to gain greater clarity on this question.

Perspectives **COMPETENCY IN MEDICAL KNOWLEDGE:** SU predicts MACE, CV death, and mortality in a contemporary guideline-treated cohort of patients with chronic coronary artery disease. IL-1β blockade with canakinumab does not affect the magnitude of these associations, while baseline kidney function and prior gout do.

**TRANSLATIONAL OUTLOOK:** SU may serve as a therapeutic target for prevention of atherosclerotic events, but additional randomized, placebo-controlled clinical trials of urate-lowering therapy in patients with hyperuricemia and atherosclerosis would be needed first.

## Funding Support and Author Disclosures

CANTOS was funded by Novartis Pharmaceutical Company. Dr Adamstein has received reimbursement for conference-related expenses from Novo Nordisk. Jean MacFadyen has received salary support from Novartis as part of a grant to BWH for work on the CANTOS trial. Dr Weber has received personal fees from Novo Nordisk, BMS, Horizon Therapeutics (now part of Amgen), and Kiniksa Pharmaceuticals (all scientific advisory board member roles) outside of the submitted work. Dr Libby is an unpaid consultant to, or involved in clinical trials for Amgen, AstraZeneca, Baim Institute, Beren Therapeutics, Esperion Therapeutics, Genentech, Kancera, Kowa Pharmaceuticals, Medimmune, Merck, Moderna, Novo Nordisk, Novartis, Pfizer, and Sanofi-Regeneron; is a member of the scientific advisory board for Amgen, Caristo Diagnostics, Cartesian Therapeutics, CSL Behring, DalCor Pharmaceuticals, Dewpoint Therapeutics, Eulicid Bioimaging, Kancera, Kowa Pharmaceuticals, Olatec Therapeutics, Medimmune, Novartis, PlaqueTec, TenSixteen Bio, Soley Thereapeutics, and Xbiotech, Inc; his laboratory has received research funding in the last 2 years from Novartis, Novo Nordisk, and Genentech. He is on the Board of Directors of Xbiotech, Inc; has a financial interest in Xbiotech, a company developing therapeutic human antibodies, in TenSixteen Bio, a company targeting somatic mosaicism and clonal hematopoiesis of indeterminate potential (CHIP) to discover and develop novel therapeutics to treat age-related diseases, and in Soley Therapeutics, a biotechnology company that is combining artificial intelligence with molecular and cellular response detection for discovering and developing new drugs, currently focusing on cancer therapeutics; his interests were reviewed and are managed by Brigham and Women’s Hospital and Mass General Brigham in accordance with their conflict of interest policies; and he has received funding support from the National Heart, Lung, and Blood Institute (1R01HL134892, 1R01HL163099-01, R01AG063839, and R01HL151627), the RRM Charitable Fund, and the Simard Fund. Dr Solomon has received salary support from CorEvitas, Janssen, and Novartis for unrelated research contracts to Brigham and Women’s Hospital. Dr Ridker has received institutional research grant support from Kowa, Novartis, Amarin, Pfizer, Esperion, Novo Nordisk, and the NHLBI; has served as a consultant to Novartis, Flame, Agepha, Ardelyx, AstraZeneca, Janssen, Civi Biopharm, Glaxo Smith Kline, SOCAR, Novo Nordisk, Health Outlook, Montai Health, Eli Lilly, New Amsterdam, Boehringer Ingelheim, RTI, Zomagen, Cytokinetics, Horizon Therapeutics, and Cardio Therapeutics; has minority shareholder equity positions in Uppton, Bitteroot Bio, and Angiowave; and has received compensation for service on the Peter Munk Advisory Board (University of Toronto), the Leducq Foundation, Paris FR, and the Baim Institute (Boston, MA).
