# Supplementary material for: Associations of Serum Urate and Cardiovascular Events in a Clinical Trial of Interleukin-1β Blockade
Source: JACC Adv. 2025 Jan 24;4(3):101583. doi: 10.1016/j.jacadv.2024.101583 (PMC11803220; doi:10.1016/j.jacadv.2024.101583)
Supplement: Supplementary Data [file mmc1.docx]

**Supplemental Table 1. Baseline characteristics by randomization group.**

|  | **Placebo** | **Canakinumab 50 mg** | **Canakinumab 150 mg** | **Canakinumab 300 mg** | **All Canakinumab Doses** | **All Participants** |
| --- | --- | --- | --- | --- | --- | --- |
| **n** | 3344 | 2170 | 2284 | 2263 | 6717 | 10061 |
| **Age** | 61 (54-68) | 61 (54-68) | 61 (55-68) | 61 (54-68) | 61 (54-68) | 61 (54-68) |
| **Female sex** | 865 (25.9%) | 541 (24.9%) | 575 (25.2%) | 606 (26.8%) | 1722 (25.6%) | 2587 (25.7%) |
| **Current smoking** | 765 (22.9%) | 531 (24.5%) | 534 (23.4%) | 536 (23.7%) | 1601 (23.8%) | 2366 (23.5%) |
| **Body mass index** | 29.7 (26.6-33.8) | 29.9 (26.6-33.9) | 29.8 (26.5-33.7) | 29.8 (26.5-33.8) | 29.8 (26.6-33.8) | 29.8 (26.6-33.8) |
| **Hypertension** | 2644 (79.1%) | 1751 (80.7% | 1814 (79.4%) | 1799 (79.5%) | 5364 (79.9%) | 8008 (79.6%) |
| **Diabetes** | 1333 (39.9%) | 854 (39.4%) | 954 (41.8%) | 888 (39.2%) | 2696 (40.1%) | 4029 (40.0%) |
| **Gout** | 250 (7.48%) | 155 (7.15%) | 175 (7.66%) | 182 (8.04%) | 512 (7.6%) | 762 (7.6%) |
| **Alcohol use (≥1 per day)** | 135 (4.04%) | 85 (3.92%) | 81 (3.55%) | 94 (4.16%) | 260 (3.9%) | 395 (3.9%) |
| **Daily exercise** | 585 (18.2%) | 364 (17.4%) | 389 (17.7%) | 379 (17.3%) | 1132 (17.4%) | 1717 (17.7%) |
| **Diuretic use** | 1205 (36.1%) | 757 (34.9%) | 853 (37.4%) | 805 (35.6%) | 2415 (36.0%) | 3620 (36.0%) |
| **Statin use** | 3044 (91.1%) | 1989 (91.7%) | 2065 (90.4%) | 2057 (90.9%) | 6112 (91.1%) | 9156 (91.0%) |
| **Urate lowering therapy** | 238 (7.12%) | 147 (6.78%) | 163 (7.14%) | 155 (6.85%) | 465 (6.92%) | 703 (6.99%) |
| **eGFR (mL/min)** | 79 (65-93) | 79 (64-92) | 79 (64.5-93) | 78 (64-93) | 78.5 (64.0-93.0) | 79.0 (64.0-93.0) |
| **SU (mg/dL)** | 6.05 (5.10-7.20) | 6.22 (5.20-7.23) | 6.20 (5.21-7.23) | 6.10 (5.20-7.23) | 6.20 (5.21-7.23) | 6.10 (5.20-7.23) |
| **hsCRP (mg/L)** | 4.10 (2.75-6.85) | 4.25 (2.80-7.15) | 4.25 (2.85-7.05) | 4.15 (2.85-7.15) | 4.20 (2.80-7.10) | 4.20 (2.80-7.05) |
| **IL-6 (ng/L)** | 2.61 (1.80-4.06) | 2.53 (1.80-4.17) | 2.56 (1.74-4.11) | 2.59 (1.79-4.08) | 2.56 (1.77-4.13) | 2.58 (1.78-4.10) |
| **Total cholesterol (mg/dL)** | 161 (137-190) | 159 (136-189) | 159 (136-188) | 161 (137-189) | 160 (136-189) | 160 (136-189) |
| **LDL cholesterol (mg/dL)** | 82.8 (64.2-107.5) | 81.2 (62.3-106) | 82.4 (63.4-106) | 83.5 (64.0-108) | 82.0 (63.0-106.7) | 82.4 (63.4-107.1) |
| **HDL cholesterol (mg/dL)** | 44.5 (37.1-52.6) | 43.7 (37.0-52.2) | 43.7 (36.3-52.0) | 44.0 (36.7-53.0) | 43.7 (36.7-52.2) | 44.0 (37.0-52.2) |
| **Triglycerides (mg/dL)** | 139 (100-194) | 140 (102-198) | 139 (101-196) | 138 (103-194) | 139 (102-196) | 139 (102-195) |

Numerical variables expressed as median (IQR). Categorical variables expressed as N (%). N, number; eGFR, estimated glomerular filtration rate; SU, serum urate; hsCRP, high-sensitivity C-reactive protein; IL, interleukin; LDL, low-density lipoprotein; HDL, high-density lipoprotein.

**Supplemental Table 2. Sensitivity analyses.**

| **Model** | **MACE** | **Cardiovascular Death** | **All-Cause Mortality** |
| --- | --- | --- | --- |
| **Univariable model** | 1.24 (1.14-1.34; <.0001) | 1.54 (1.38-1.72; <.0001) | 1.48 (1.36-1.62;<.0001) |
| **Inclusive Multivariable model** | 1.07 (0.98-1.17; 0.12) | 1.29 (1.14-1.46; <.0001) | 1.24 (1.13-1.37; <.0001) |
| **Multivariable minus age** | 1.03 (0.95-1.13; 0.48) | 1.21 (1.07-1.37; 0.002) | 1.16 (1.05-1.28; 0.003) |
| **Multivariable minus sex** | 1.12 (1.03-1.23; 0.009) | 1.33 (1.18-1.50; <.0001) | 1.29 (1.17-1.42; <.0001) |
| **Multivariable minus BMI** | 1.07 (0.98-1.17; 0.15) | 1.24 (1.10-1.41; 0.0006) | 1.21 (1.10-1.34; 0.0001) |
| **Multivariable minus smoking status** | 1.06 (0.97-1.16; 0.19) | 1.28 (1.13-1.45; <.0001) | 1.24 (1.12-1.36; <.0001) |
| **Multivariable minus hypertension** | 1.07 (0.98-1.18; 0.12) | 1.29 (1.14-1.46; <.0001) | 1.24 (1.13-1.37; <.0001) |
| **Multivariable minus type 2 diabetes** | 1.07 (0.98-1.18; 0.12) | 1.29 (1.14-1.46; <.0001) | 1.24 (1.13-1.37; <.0001) |
| **Multivariable minus LDL-c** | 1.09 (0.99-1.19; 0.07) | 1.28 (1.13-1.45; <.0001) | 1.24 (1.12-1.37; <.0001) |
| **Multivariable minus hsCRP** | 1.08 (0.98-1.18; 0.11) | 1.29 (1.14-1.46; <.0001) | 1.25 (1.13-1.38; <.0001) |
| **Multivariable minus eGFR** | 1.12 (1.03-1.22; 0.01) | 1.35 (1.20-1.52; <.0001) | 1.31 (1.20-1.44; <.0001) |
| **Multivariable minus diuretic use** | 1.13 (1.03-1.23; 0.008) | 1.41 (1.25-1.60; <.0001) | 1.34 (1.21-1.47; <.0001) |
| **Multivariable minus urate lowering therapy** | 1.07 (0.98-1.17; 0.12) | 1.28 (1.13-1.45; 0.0001) | 1.23 (1.12-1.36; <.0001) |
| **Multivariable minus daily alcohol use** | 1.07 (0.98-1.17; 0.12) | 1.29 (1.14-1.46; <.0001) | 1.25 (1.13-1.37; <.0001) |
| **Multivariable minus daily exercise** | 1.07 (0.98-1.17; 0.13) | 1.28 (1.13-1.45; <.0001) | 1.24 (1.13-1.37; <.0001) |

Sensitivity analysis performed with one covariable removed from the inclusive multivariable model at a time. Hazard ratios shown are for trend across baseline SU groups for MACE, cardiovascular death, and all-cause mortality. Inclusive multivariable model adjusted for treatment arm, age, sex, BMI, smoking status, hypertension, type 2 diabetes, LDL-c, hsCRP, eGFR, diuretic use, urate lowering therapy, daily alcohol use, and daily exercise. Data shown as HR (95% CI; P). SU, serum urate; BMI, body mass index; LDL-c, low-density lipoprotein cholesterol; hsCRP, high-sensitivity C-reactive protein; eGFR, estimated glomerular filtration rate; MACE, major adverse cardiovascular events; CV, cardiovascular.

**Supplemental Table 3. Risk of MACE, CV death, and all-cause mortality depending on prior or incident gout.**

|  | **No baseline gout** | **Baseline gout** | **No incident gout flare** | **Incident gout flare** |
| --- | --- | --- | --- | --- |
| **n** | 9299 | 762 | 9953 | 108 |
| **MACE** |  |  |  |  |
| Incidence | 1335 (14.4%) | 155 (20.3%) | 1315 (14.3%) | 20 (18.5%) |
| HR (95% CI; P) | 1 (ref) | 1.46 (1.23-1.72; <.0001) | 1 (ref) | 1.22 (0.79-1.90; 0.37) |
| **CV Death** |  |  |  |  |
| Incidence | 603 (6.5%) | 64 (8.4%) | 593 (6.5%) | 10 (9.3%) |
| HR (95% CI; P) | 1 (ref) | 1.31 (1.01-1.70; 0.04) | 1 (ref) | 1.35 (0.72-2.52; 0.35) |
| **All-cause mortality** |  |  |  |  |
| Incidence | 966 (10.4%) | 114 (15.0%) | 952 (10.4%) | 14 (13.0%) |
| HR (95% CI; P) | 1 (ref) | 1.45 (1.20-1.76; 0.0002) | 1 (ref) | 1.16 (0.69-1.97; 0.57) |

Incidence reported as the number who experienced an event (percent of total in that column). Hazard ratios compared participants with gout at baseline to those without gout at baseline (reference), and participants who developed new gout to those who did not (reference). These data were expressed as HR (95% CI; P-value). N, number; MACE, major adverse cardiovascular events; HR, hazard ratio; CI, confidence interval; CV, cardiovascular.

**Supplemental Table 4. Serum Urate Correlations.**

|  | **Correlation with SU (ρ(P))** | | |
| --- | --- | --- | --- |
| **Group** | **Gout** | **No gout** | **All Participants** |
| **n** | 762 | 9299 | 10061 |
| **Age** | -0.07 (P=0.047) | 0.05 (P<.0001) | 0.04 (P<.0001) |
| **Body mass index** | 0.02 (P=0.56) | 0.14 (P<.0001) | 0.14 (P<.0001) |
| **eGFR** | -0.16 (P<.0001) | -0.31 (P<.0001) | -0.31 (P<.0001) |
| **HbA1C** | -0.005 (P=0.89) | -0.05 (P<.0001) | -0.04 (P<.0001) |
| **LDL cholesterol** | 0.11 (P=0.003) | 0.02 (P=0.045) | 0.02 (P=0.01) |
| **HDL cholesterol** | -0.05 (P=0.16) | -0.10 (P<.0001) | -0.10 (P<.0001) |
| **Total cholesterol** | 0.12 (P=0.001) | 0.04 (P=0.0002) | 0.04 (P<.0001) |
| **Triglycerides** | 0.10 (P=0.004) | 0.15 (P<.0001) | 0.15 (P<.0001) |
| **hsCRP** | 0.07 (P=0.07) | 0.06 (P<.0001) | 0.07 (P<.0001) |
| **IL-6** | 0.02 (P=0.73) | 0.08 (P<.0001) | 0.08 (P<.0001) |
| **IL-18** | 0.007 (P=0.89) | 0.08 (P<.0001) | 0.08 (P<.0001 |
| **WBC** | -0.009 (P=0.81) | -0.01 (P=0.27) | -0.01 (P=0.25) |
| **ANC** | -0.03 (P=0.45) | -0.01 (P=0.66) | -0.005 (P=0.59) |
| **ALC** | 0.03 (P=0.39) | -0.04 (P=0.0003) | -0.04 (P=0.0002) |
| **NLR** | -0.05 (P=0.14) | 0.03 (P=0.0044) | 0.03 (P=0.006) |
| **Fibrinogen** | 0.08 (P=0.23) | 0.04 (P=0.09) | 0.05 (P=0.03) |

Spearman correlation coefficients comparing baseline SU and other baseline parameters in participants with and without gout. Values formatted as ρ (P). SU, serum urate; n, number; eGFR, estimated glomerular filtration rate; HbA1C, hemoglobin A1C; LDL, low density lipoprotein; HDL, high density lipoprotein; hsCRP, high-sensitivity C-reactive protein; IL, interleukin; WBC, white blood cells; ANC, absolute neutrophil count; ALC, absolute lymphocyte count; NLR, neutrophil-lymphocyte ratio.

**Supplemental Figure 1. Baseline serum urate in sub-groups.**


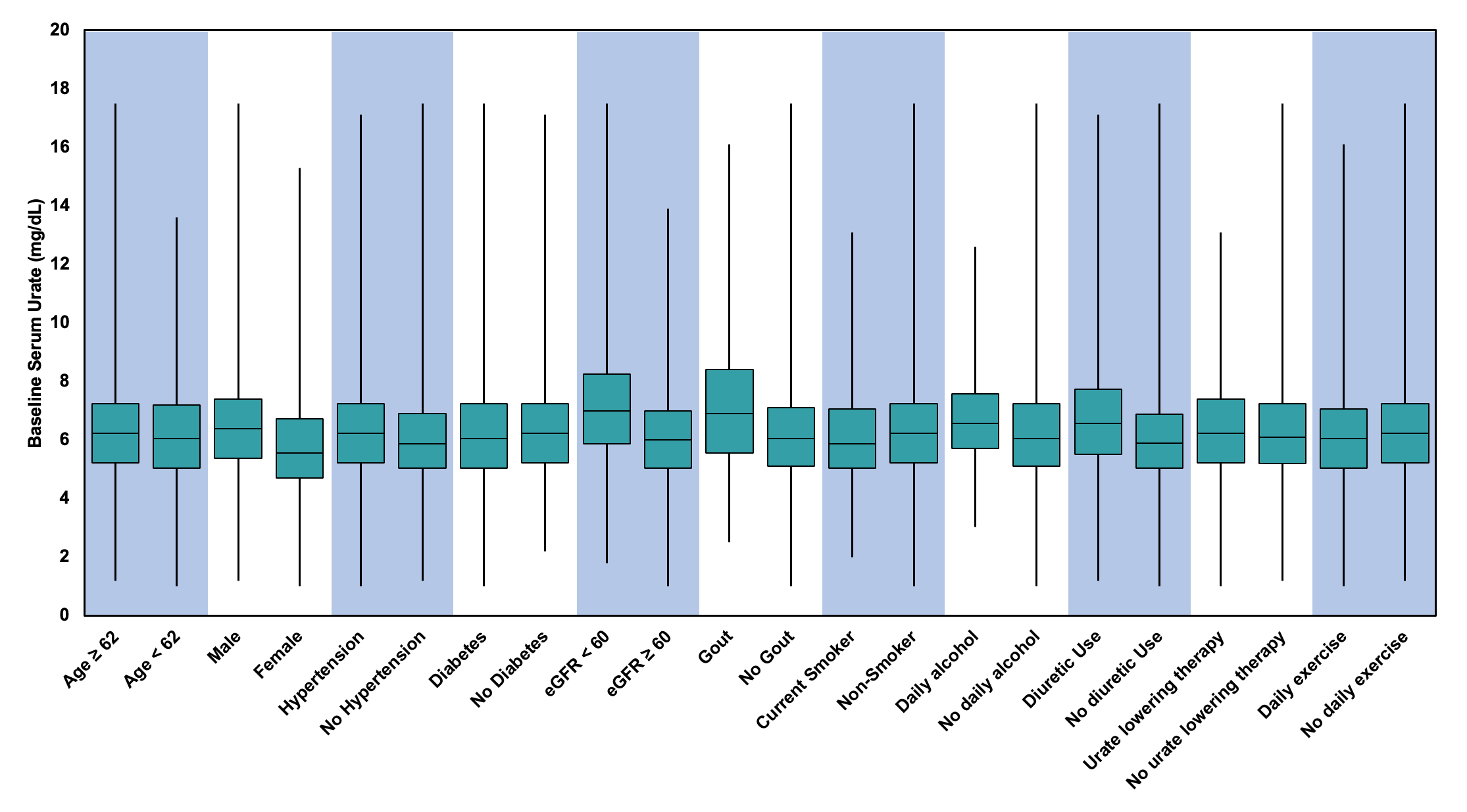
The center of the box designates the median, with the box bounded by the first and third quartiles, and the whiskers reaching to the extreme values. eGFR, estimated glomerular filtration rate.

**Supplemental Figure 2. Serum urate over time.**


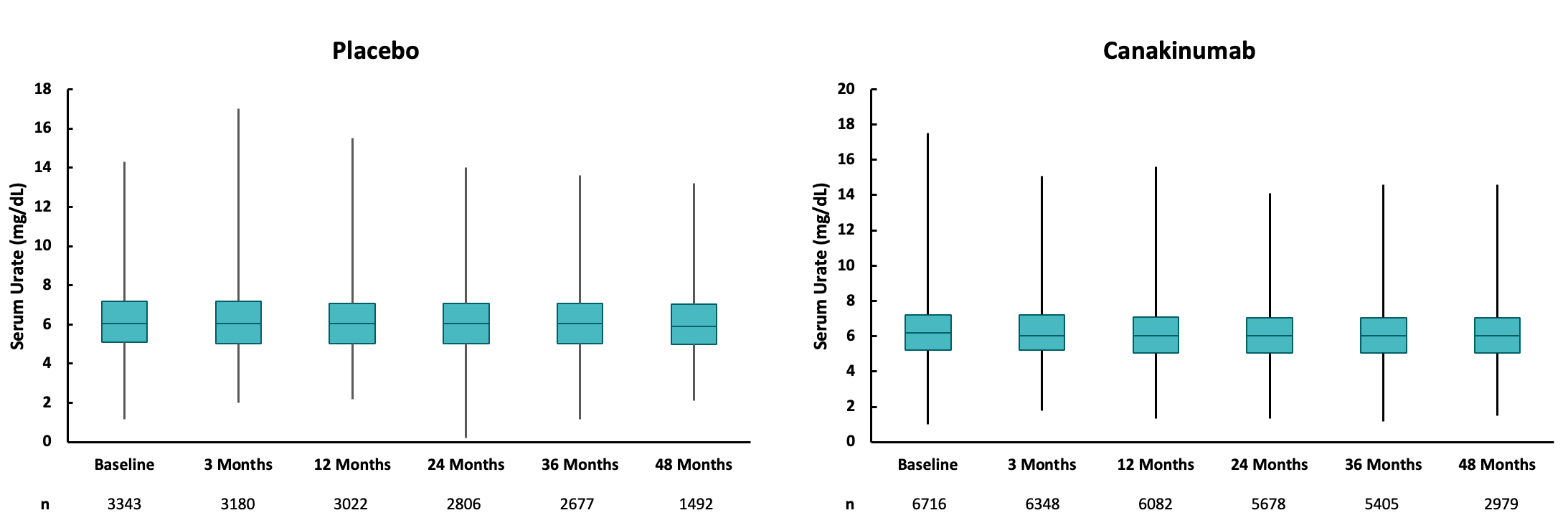


Box and whisker plots of the distribution of serum urate (mg/dL) over time in the placebo (left) and canakinumab (right) arms. The center of the box designates the median, with the box bounded by the first and third quartiles, and the whiskers reaching to the extreme values. n, number.
